# The PODCAST-question: postoperative dilation versus calibration after surgical treatment of Hirschsprung’s disease—what is necessary?

**DOI:** 10.1007/s00383-026-06351-4

**Published:** 2026-02-27

**Authors:** Daniel Keese, Florian Friedmacher, Udo Rolle, Hannah Neeser, Tim Gerwinn, Sasha Job Tharakan, Ueli Möhrlen

**Affiliations:** 1 Department of Pediatric Surgery, University Children’s Hospital Zurich, Lenggstrasse 30, 8008 Zurich, Switzerland; 2 Department of Pediatric Surgery and Pediatric Urology, Johannes Wolfgang Goethe University Frankfurt, Theodor-Stern-Kai 7, 60590 Frankfurt, Germany

**Keywords:** Hirschsprung disease, Anal dilation, Anastomotic stricture, Hirschsprung associated enterocolitis, Postoperative management

## Abstract

**Purpose:**

Postoperative anal dilation after surgery for Hirschsprung disease (HD) still remains common practice. This study aimed to evaluate the benefit of routine dilations compared with a single postoperative calibration.

**Methods:**

Patients who underwent HD surgery within the first year of life at the University Children’s Hospital Zurich or the University Hospital Frankfurt were retrospectively reviewed. Two groups were compared: those with serial postoperative dilations (PD) and those with a single calibration (PC). Primary outcomes included anastomotic stricture, Hirschsprung-associated enterocolitis (HAEC), and constipation/fecal incontinence.

**Results:**

A total of 91 patients were included—46 (51%) in the PD group and 45 (49%) in the PC group. Strictures occurred in 5 patients (10.9%) with PD and 9 (20%) with PC (p = 0.259). HAEC developed in 5 (10.9%) PD and 6 (13.3%) PC patients (p = 0.718). Rates of constipation/fecal incontinence were similar (p = 0.67).

**Conclusion:**

Routine postoperative anal dilation after HD surgery does not significantly reduce the incidence of strictures, HAEC, or constipation/fecal incontinence. Single postoperative calibration appears sufficient to determine whether further dilation is necessary.

## Introduction

The key component in the treatment of Hirschsprung’s disease (HD) is surgery. The primary objectives of surgery are to resect the aganglionic segment of the rectum and colon, to pull down the ganglionic bowel for an anastomosis to the anus, as well as to preserve the nervous and muscular structures of the pelvic floor. Several different surgical techniques for treating HD exist, varying in their approach and adapted from the original concepts of Swenson, Duhamel, and Soave–Boley [1–3]. The advancement of minimally invasive and single-stage surgical techniques has led to an increasing shift away from open access to the abdominal cavity [4]. In many clinics, transanal pull-through surgery has become the method of choice [5]. Depending on the length of the aganglionic segment, additional laparoscopic or open bowel mobilization may be necessary. Postoperative complications may include anastomotic stricture, anastomotic leakage, wound infection, HAEC, persistent constipation or fecal incontinence [6]. As anastomotic strictures can significantly reduce the long-term functional quality of life, most surgeons therefore implement regular postoperative anal dilation protocols hoping to prevent this [7]. The concept of preventing anastomotic strictures with a postoperative anal dilation protocol was primarily set forward by Levitt and Pena for patients who underwent posterior sagittal anorectoplasty (PSARP) for anorectal malformations (ARM). Despite the difference in underlying pathology, anatomy and operative technique, the majority of pediatric surgeons adhere to Pena’s postoperative protocol for PSARP patients when treating HD patients. Typically, anal dilations are continued for a duration up to 8 months after surgery [8]. However, as of yet, there is no conclusive evidence that these postoperative prophylactic anal dilations actually reduce the anastomotic stricture rate after surgery in HD patients. Therefore, the aim of this retrospective multicenter cohort study is to evaluate the benefit of prophylactic anal dilations compared to single calibration after surgery in HD patients by comparing the incidence of postoperative complications. The outcomes that were of primary interest in this study were the development of anastomotic stricture and the occurrence of HAEC.

## Methods

### Data collection

This study involves a retrospective data analysis, utilizing previously collected patient data from the patient record of children with HD from two different clinics (University Children’s Hospital Zurich, Switzerland and University Hospital Frankfurt am Main, Germany). The local Ethics Committee of Zurich approved the study protocol (BASEC Nr. 2023–01511).

Patients were included if they underwent definitive surgery for HD within the first year of life at one of the two participating centers between January 2009 and December 2019. Eligible surgical techniques comprised Soave-based pull-through procedures, including transanal endorectal pull-through (TERPT, De la Torre technique [9]), as well as Swenson, Duhamel, and laparoscopically assisted pull-through procedures.

Patients were excluded if they were older than 1 year at the time of surgery or if there was a documented refusal or withdrawal of consent for the secondary use of health-related data (general consent). Those with inadequate follow-up were also excluded. In addition, children with severe syndromic diseases were excluded due to their known association with less favorable outcomes following pull-through procedures, which could have introduced significant confounding effects [10].

To ensure a sufficiently long follow-up period for the younger patients included in the study, data collection continued until December 2023. The collected data included demographic variables, the type of operation performed, length of the aganglionic segment and postoperative complications (anastomotic stricture, HAEC, constipation and/or fecal incontinence). The operative protocols and the pathology reports were used to determine the length of the aganglionic bowel segment.

For the purposes of this study anastomotic stricture was defined as present if the age-appropriate sized Hegar dilator could not be advanced through the anal canal and/or if a narrow ring was palpable by the surgeon during rectal examination. HAEC was defined based on the guidelines released in by the American Pediatric Surgical Association (APSA) in 2017 [11]. Bowel function was assessed using standardized definitions for constipation and fecal incontinence, with stool consistency evaluated using the Bristol Stool Scale. In particular constipation was defined as present if the documented symptoms fulfilled the definition of constipation established by the Paris Childhood Constipation Terminology Group (PACCT) [12]. The presence of fecal incontinence was determined by the occurrence of voluntary or involuntary passage of feces outside of toilet-trained contexts in children aged four years or older also according to the PACCT [9].

### Postoperative anal dilation group (PD)

Children who underwent surgery at the Children’s Hospital Zurich received regular postoperative anal dilation (PD group). The first postoperative dilation is performed by the surgeon on postoperative day 8 to 10 in an in- or outpatient setting. The size of the largest dilator used is based on the patient’s age as described by Pena [13]. Regardless of whether a stricture is detected during the first dilation, parents are taught to continue regular dilations. The size of the Hegar dilator is slowly increased over several weeks—according to a standard protocol until at least the 12th postoperative week (see Table 1). After completion of the dilations patients continue to visit our clinic on average every 6–12 months.

**Table 1:** Dilation treatment after pull-through surgery for HD in the PD group

Hegar size				Frequence	Duration (weeks)
Age: ≤ 4 months	≤ 8 months	≤ 12 months	1–3 years		
6–10	7–11	8–12	9–13	1 × daily	1/2
8–11	9–12	10–13	11–14	1 × daily	1/2
9–12	10–13	11–14	12–15	1 × daily	3
9–12	10–13	11–14	12–15	every 2nd day	2
Clinical check-up after 6 weeks					
9–12	10–13	11–14	12–15	every 2nd day	2
9–12	10–13	11–14	12–15	2 × weekly	2
9–12	10–13	11–14	12–15	1 × weekly	2

Clinical check-up after 12 weeks (dilation treatment should then usually be terminated)

### Postoperative calibration group (PC)

Children who underwent surgery at the University Hospital Frankfurt am Main received a single anal calibration on the 8th-10th postoperative day (PC group). The size of the dilator used for calibration was also chosen based on the patient’s age as described by Pena [13]. In this group, if an anastomotic stricture was subsequently diagnosed, anal dilations were initiated according to a internal protocol. Patients were thereafter seen regularly in the outpatient clinic every 6 to 12 months.

### Statistical methods

The collected data were analyzed using SPSS software version 18 (SPSS Inc. Released 2009. PASW Statistics for Windows, Version 18.0. Chicago: SPSS Inc). The continuous data were expressed as the median and interquartile range (IQR), whereas the categorical data were expressed as numbers and percentages. For the qualitative data, the chi-square test was used to compare the difference in proportions between independent groups. When the expected counts were less or equal than 5, Fisher’s exact test was used. For the quantitative data, the Mann–Whitney U test was used to compare the mean between each group. A p-value of p < 0.05 was statistically significant.

## Results

### Demographics

A total of 91 patients with HD were included in this study, with 46 patients (51%) in the PD group and 45 (49%) in the PC group. The demographic data of both groups are shown in Table 2. The two groups show similar characteristics in term of gender distribution, birth weight, age at surgery and the type of operation. Also follow-up duration was comparable between the two groups, with a median age at last follow-up of 7 years in both groups. However, the age at diagnosis was significantly lower and the length of the resected aganglionic segment was significantly longer in the PD group.

**Table 2:** Patients’ demographic data: comparison between the PD group and PC group

	PD group (n = 46)	PC group (n = 45)	Total (n = 91)	p-value
Gender, n (%)				0.217
Male	34 (73.9%)	38 (84.4%)	72 (79.1%)	
Female	12 (26.1%)	7 (15.6%)	19 (20.9%)	
Birth weight (median g (IQR))	3280 (790)	3400 (440)	3315 (547.5)	0.289
Age at diagnosis (median weeks (IQR))	2 (3)	8 (19)	4 (10.8)	0.05
Age at surgery (median months (IQR))	4 (2)	5 (4)	4 (3)	0.116
Operation method, n (%)				0.183
TERPT	38 (82.6%)	33 (73.3%)	71 (78%)	
TERPT including laparotomy / laparoscopic-assisted	8 (17.4%)	9 (20.0%)	17 (18.7%)	
Other / unspecified technique	–	3 (6.7%)	3 (3.3%)	
Length of aganglionic segment (median cm (IQR))	35 (17)	19 (15)	28 (22)	0.05
Age at last follow-up (median years (IQR))	7 (6)	7 (4)	7 (5)	0.204

*IQR* interquartile range, *g* gram

### Postoperative stricture

Comparing the incidences of postoperative stricture between the two groups, 5 patients (10.9%) of the PD group and 9 patients (20%) of the PC group developed a stricture (p = 0.259) (Table 3). In the PD group, the diagnosis of the first stricture for the five patients was made between 22 and 356 days postoperatively. All patients had a TERPT according to De La Torre technique. Two of these patients needed intraabdominal mobilization by laparotomy (Patient No 3) or laparoscopy (Patient No 5) (see Table 4). None of these five patients required re-operation, however dilations were continued for a longer period of time and were restarted in two patients. In the PC group, the diagnosis of stricture was made in 9 patients between day 13 and day 1623 after surgery. Excluding one patient all others had a TERPT procedure (see Table 5). All patients were successfully managed with initial dilation with or without general anesthesia followed by a period of daily or weekly dilation by the parents. No additional surgery was necessary. There was no significant difference between the two groups (p = 0.645) regarding time of developing a postoperative stricture.

**Table 3:** Comparison of the incidences of postoperative complications between the PD and PC group

	PD group (n = 46)	PC group (n = 45)	Total (n = 91)	p-value
Stricture; n (%)	5 (10.9%)	9 (20%)	14 (15.4%)	0.259
HAEC; n (%)	5 (10.9%)	6 (13.3%)	11 (12.1%)	0.758
Constipation / fecal incontinence (n; %)	16 (34.8%)	14 (32.6%)	30 (32.9%)	0.8

**Table 4:** Patients of the PD group with postoperative stricture and HAEC

Patient no	Gestational age	Birth weight (g)	Age at operation (months)	Operation method	Length of resected bowel segment	Time of stricture (days after surgery)	Total no of HAEC	Time of first HAEC (months after surgery)
1	37 4/7	–	5	TERPT	35 cm	61	–	–
2	41 3/7	4120	3	TERPT	15 cm	42	–	–
3	38 2/7	2860	3	TERPT including laparotomy	45 cm	159	–	–
4	31 6/7	1360	5	TERPT	15 cm	22	–	–
5	38 4/7	3070	3	TERPT with laparoscopic assistance	40 cm	356	–	–
6	39 5/7	3180	7	TERPT including laparotomy	50 cm (TCA)	–	4	40
7	40	–	7	TERPT	50 cm (TCA)	–	3	28
8	40	2480	4	TERPT	31 cm	–	1	7
9	35 1/7	2600	4	TERPT	40 cm	–	1	11
10	38 1/7	3085	3	TERPT	35 cm	–	1	1

*g* gram, *TCA* total colonic aganglionosis; no number

**Table 5:** Patients of the PC Group with postoperative stricture and HAEC

Patient no	Gestational age	Birth weight (g)	Age at operation (months)	Operation method	Length of resected bowel segment	Time of stricture (days after surgery)	Total no of HAEC	Time of first HAEC (months after surgery)
1	38 1/7	2730	4	TERPT	9 cm	53	–	–
2	35 2/7	1800	3	TERPT	21 cm	43	–	–
3	40 3/7	3300	7	TERPT	23 cm	44	–	–
4	39 6/7	3150	3	TERPT	30 cm	15	–	–
5	37 2/7	2750	3	TERPT	30 cm	35	–	–
6	37 3/7	3450	3	TERPT	13 cm	13	1	5
7	37 1/7	2660	1	TERPT	21 cm	1623	3	53
8	37 0/7	3100	2	TERPT	30 cm	48	–	–
9	38 3/7	3580	2	Other	80 cm (TCA)	75	–	–
10	38 5/7	2860	5	TERPT	15 cm	–	1	7
13	40 2/7	3000	3	TERPT	26 cm	–	2	25
14	38 3/7	3580	2	Other	80 cm (TCA)	–	1	27
15	39 5/7	3450	5	Other	50 cm (TCA)	–	1	18

*g* gramm, *TCA* total colonic aganglionosis; no number

A non-inferiority test was conducted to compare the single calibration method (PC group) to regular postoperative dilations (PD group) based on postoperative stricture rates. After performing Fisher’s Exact test to assess the independence between the two groups, non-inferiority was established when the lower bound of the 95% one-sided confidence interval (CI) for the difference in stricture rates between the groups was below the predefined margin of 10%. The difference in stricture rates between the two groups was 9.1%. The one-sided 95% CI for the difference (0.0018, 0.0861) was below the predefined non-inferiority margin of 10%, confirming that a single postoperative calibration is not significantly worse than regular postoperative dilations. To further evaluate the clinical impact of avoiding regular postoperative dilations, the number needed to treat (NNT) was calculated. The NNT was determined to be 11, indicating that 11 patients in the PD group would need to be treated to prevent one case of stricture.

### Postoperative HAEC

Regarding the incidence of HAEC after surgery 5 patients (10.9%) in the PD group and 6 patients (13.3%) in the PC group developed HAEC (p = 0.718) postoperatively (see Table 3).

Two of the five patients with postoperative HAEC in the PD group had a total colonic aganglionosis (TCA). These two patients had recurrent episodes of HAEC during the follow up period. The other three patients had a long aganglionic segment measuring more than 30 cm. In addition, the two patients with a TCA showed a later onset of HAEC (28 and 40 months after surgery) compared to the other three patients (1, 7 and 11 months after surgery). All patients underwent the same surgical procedure, a TERPT (see Table 4).

Similarly, two of the 6 patients in the PC group, had a TCA. However, both patients had only one episode of HAEC. One patients had two and one had three episodes of HAEC. Four patients underwent a TERPT procedure, the operation method of the two patients with TCA was straight ileoanal anastomosis without forming a pouch. In these cases the specific surgical technique was not named. (see Table 5).

When comparing timing of HAEC after surgery, no relevant difference (p = 0.928) was observed in the PD group (mean 17 months) compared to the PC (mean 22 months). In the PC group 2 patients with postoperative stricture also developed HAEC (Patient No 6 and 7 in Table 5), while no patient in the PD group had a combined stricture and HAEC.

Since demographic differences existed between the PD and PC groups regarding median age at diagnosis and length of the aganglionic segment, these variables were considered potential confounders. Therefore, an analysis of covariance was performed to assess their influence on postoperative stricture rate and HAEC. Neither age at diagnosis nor length of the aganglionic segment was significantly associated with the occurrence of stricture or postoperative HAEC (Table 6). Given the lack of significant association, no additional adjustment was performed.

**Table 6:** Analysis of covariance evaluating the influence of age at diagnosis and length of the aganglionic segment on postoperative stricture and HAEC

Variable	Stricture (p)	HAEC (p)
Age at diagnosis	0.401	0.395
Length of aganglionic segment	0.764	0.126

### Constipation and fecal incontinence

A secondary endpoint was to assess the effects of not routinely performing postoperative anal dilations, focusing on constipation and fecal incontinence. In our cohorts, both groups showed similar results: 16 patients (34.8%) in the PD group and 14 patients (32.6%) in the PC group had constipation and and/or fecal incontinence postoperatively (see Table 3). All patients were treated with laxatives or bowel irrigation.

## Discussion

This is the first retrospective multi-center cohort study to compare two different approaches to postoperative anal dilations after HD surgery in respect to anastomotic stricture, HAEC and constipation / fecal incontinence. We demonstrate that a single postoperative anal calibration is not inferior to postoperative regular anal dilations.

Regarding the operation techniques, the functional outcomes for HD can vary significantly. Transanal approaches have shown significant advantages over open surgery in reducing the incidence of soiling, incontinence, and constipation; however, most studies show no difference in the incidence of postoperative HAEC or anastomotic strictures [14].

In patients with proven anastomotic strictures, serial anal dilations have been shown to be effective in treating the strictures and avoiding further surgical management [7]. However, in the absence of anastomotic stricture, the benefit of performing routine anal dilations remains uncertain. In 2004, Aworanti demonstrated that routine anal dilations did not result in a reduction in the incidence of anastomotic strictures or HAEC [15]. Similarly, in a current study, Beati et al. suggest that patients treated with transanal endorectal pull-through for HD may not require routine postoperative anal dilations—and beyond that not even a single calibration—to prevent postoperative strictures or HAEC [16]. Both studies were single center studies with a limited number of patients and a short follow-up. However, other authors suggest that prophylactic dilations, as proposed by Lewitt and Pena for patients with ARM following PSARP, may prevent postoperative anastomotic strictures [6, 17, 18]. In relation to the frequency of dilation, Temple et al. [7] reported that weekly dilations of the anastomosis by a surgeon has a similar incidence of anastomotic strictures and postoperative HAEC to cases involving daily anal dilation by parents.

Furthermore, performing anal dilations can be a significant source of stress for the parents. They usually have to hold down their child for the dilations, which parents can perceive as unpleasant or painful for the child. Consequently, routine anal dilations are a controversial topic, not only in regard to potential physical, but also psychological harm they may cause. Vandvik et al. [[19]] describe several issues in this context, such as a double-bind conflict between the child and the parent who performs the dilation.

Anastomotic stricture is considered one of the most important postoperative complications after HD surgery and varies from 3 to 35% [6, 20, 21]. It may result from ischemia at the anastomosis, excessive tension, incomplete suturing technique, or ischemia of a rectal muscular cuff [20]. The total stricture rate of 15.4% in our cohorts is comparable to recent literature6. Both the PD and the PC group have similar rates of anastomotic stricture suggesting that routine anal dilation after surgery may not prevent postoperative stricture. One of the notable differences between our cohorts, was one patient in the PC group who developed a stricture after 4 years (1623 days). This is an unusually late diagnosis of a stricture and may well have been missed during the clinical follow-up. Aworanti hypothesized that routine dilations may not affect the overall stricture rate, but appear to have prevented symptomatic early strictures [15]. However, if we exclude this patient with the late stricture, our cohorts have a similar duration to stricture development. Furthermore, we did not find any confounding factors, such as age at diagnosis or length of the aganglionic segment, that put patients at a higher risk of developing an anastomotic stricture. Finally, it is important to note that none of the patients with stricture required a re-operation. Instead, all patients were successfully managed with regular dilations, occasionally performed under anesthesia.

HAEC is another feared postoperative complication after HD surgery and is known to be more common in patients with longer aganglionic segments [22]. Although up to 50% of patientsm [23] may suffer from HAEC preoperatively, high rates of postoperative HAEC up to 35% [24, 25] have also been described. The overall incidence of 12.1% of postoperative HAEC in our cohorts compares well with the literature [26, 27]. According to Hagens et al. [23] the postoperative risk of developing HAEC appears to be unaffected by either the surgical method employed or the length of the resected bowel segment. Postoperative dilation does not seem to be effective in preventing HAEC, as in our cohorts there was no significant difference in incidence of HAEC between the groups. This remains true even after correcting for confounding factors such as gestational age and time at diagnosis. Taken together with the available literature [7, 15], our results support the hypothesis that routine dilation does not reduce the risk of postoperative HAEC.

However, anastomotic stricture has been identified as a risk factor for an increased incidence of HAEC [6, 28]. In our cohort, two patients in the PC group had not only an anastomotic stricture, but also several episodes of postoperative HAEC. Both patients had the first episode of HAEC before the stricture was treated with dilations. Neither patient had another episode of HAEC after the stricture was treated.

Following HD surgery intestinal symptoms, such as constipation and fecal incontinence may persist or develop newly despite the surgical removal of the aganglionic segment. These disorders are known to have a significant impact on the physical and emotional well-being of children, adolescents, and adults with HD [29]. Both aspects – constipation and fecal incontinence—are reported in up to 71% and up to 50% of HD patients, respectively, after surgery [30, 31]. The reason for this high rate of postoperative constipation or fecal incontinence remains unclear. There is increasing evidence that structural abnormalities in the proximal, ganglionic colon (e.g. imbalanced expression of neurotransmitters and neuroproteins or abnormal expression of enteric pacemaker cells) may contribute to persistent bowel dysfunction [32]. Moreover earlier surgical intervention (preferably between 6 months and 1 year of age) is associated with better long-term fecal continence, regardless of whether an abdominal or transanal approach is used [14, 33]. There is no significant difference in postoperative continence between the two techniques. Based on our results, there was also no significant difference in symptoms of constipation and/or fecal incontinence between the two cohorts, suggesting that postoperative dilation had no effect on these outcomes.

However, postoperative bowel function was routinely assessed in both centers using standardized definitions for constipation and fecal incontinence, complemented by an evaluation of stool consistency using the Bristol Stool Scale. While these measures allowed for a structured assessment of key functional outcomes, they do not capture all relevant dimensions of postoperative bowel function and therefore represent a limitation of the study. In addition, the multicenter design may introduce institutional bias related to center-specific postoperative protocols. As both participating institutions are tertiary pediatric surgery units adhering to established Hirschsprung disease management principles, the impact of such bias is likely limited; nevertheless, it should be considered when interpreting the results.Despite these limitations, the study benefits from good generalizability, as it was conducted in two university hospitals with differing pull-through techniques and postoperative management strategies for anal dilations, thereby reflecting real-world clinical practice. The long and comparable follow-up duration (7 years) in both groups represents another strength of this study, as it minimizes follow-up–related bias and allows for a robust evaluation of late postoperative complications.

## Conclusion

Routine anal dilation after HD surgery does not reduce the rates of postoperative stricture, HAEC, constipation or fecal incontinence. A single postoperative calibration is sufficient to decide whether further regular dilations are necessary.

## Data Availability

No datasets were generated or analysed during the current study.
